# Performance of testers with contrasting provitamin A content to evaluate provitamin A maize for resistance to *Aspergillus flavus* infection and aflatoxin production

**DOI:** 10.3389/fpls.2023.1167628

**Published:** 2023-05-10

**Authors:** M. Mboup, A.O. Aduramigba-Modupe, A.-R. S. Maazou, B. Olasanmi, W. Mengesha, S. Meseka, I. Dieng, R. Bandyopadhyay, A. Menkir, A. Ortega-Beltran

**Affiliations:** ^1^ Pan African University Life and Earth Sciences Institute (including Health and Agriculture), University of Ibadan, Ibadan, Nigeria; ^2^ International Institute of Tropical Agriculture (IITA), Ibadan, Nigeria; ^3^ Department of Crop Protection and Environmental Biology, Faculty of Agriculture, University of Ibadan, Ibadan, Nigeria; ^4^ Department of Crop and Horticultural Sciences, Faculty of Agriculture, University of Ibadan, Ibadan, Nigeria

**Keywords:** aflatoxin, provitamin A, carotenoids, testers, tropical maize

## Abstract

In sub-Saharan Africa (SSA), millions of people depend on maize as a primary staple. However, maize consumers in SSA may be exposed to malnutrition due to vitamin A deficiency (VAD) and unsafe aflatoxin levels, which can lead to serious economic and public health problems. Provitamin A (PVA) biofortified maize has been developed to alleviate VAD and may have additional benefits such as reduced aflatoxin contamination. In this study, maize inbred testers with contrasting PVA content in grain were used to identify inbred lines with desirable combining ability for breeding to enhance their level of resistance to aflatoxin. Kernels of 120 PVA hybrids generated by crossing 60 PVA inbreds with varying levels of PVA (5.4 to 51.7 µg/g) and two testers (low and high PVA, 14.4 and 25.0 µg/g, respectively) were inoculated with a highly toxigenic strain of *Aspergillus flavus*. Aflatoxin had a negative genetic correlation with β-carotene (*r* = −0.29, *p* < 0.0001) and PVA (*r* = −0.23, *p* < 0.0001), indicating that hybrids with high PVA content accumulated less aflatoxin than those with low to medium PVA. Both general combining ability (GCA) and specific combining ability (SCA) effects of lines and testers were significant for aflatoxin accumulation, number of spores, PVA, and other carotenoids, with additive gene actions playing a prominent role in regulating the mode of inheritance (GCA/SCA ratio >0.5). Eight inbreds had combined significant negative GCA effects for aflatoxin accumulation and spore count with significant positive GCA effects for PVA. Five testcrosses had combined significant negative SCA effects for aflatoxin with significant positive SCA effects for PVA. The high PVA tester had significant negative GCA effects for aflatoxin, lutein, β-carotene, and PVA. The study identified lines that can be used as parents to develop superior hybrids with high PVA and reduced aflatoxin accumulation. Overall, the results point out the importance of testers in maize breeding programs to develop materials that can contribute to controlling aflatoxin contamination and reducing VAD.

## 1 Introduction

Maize (*Zea mays* L.) is one of the leading cereal crops grown across the globe and a staple in many low- and middle-income countries. It is cultivated on approximately 197 million ha, generating an annual grain yield estimated at 1,137 million tons; such large production has been realized by the widespread use of high-yielding hybrids, significant area expansion, and use of complementary inputs (FAOSTAT, 2021; Erenstein et al., 2022). Maize provides approximately 30% of the total calories for more than 4.5 billion people, and in sub-Saharan Africa (SSA), maize is an extremely important constituent of the diets of millions (Menkir et al., 2008; Shiferaw et al., 2011). In SSA, white maize is the most common type, but it is devoid of provitamin A (PVA) carotenoids (avg. 2 μg/g), while yellow maize containing carotenoids has mostly been used for animal feed and production, and consumption of orange maize (rich in PVA) is very much limited. Low consumption of PVA leads to vitamin A deficiency (VAD), which is a significant health problem in SSA and Southeast Asia (Bouis and Saltzman, 2017).

VAD, which affects up to 5.7 million preschool-age children and 9.7 million pregnant women, can cause night blindness and increase the risk of morbidity and mortality from several diseases, including anemia, measles, diarrhea, and malaria (World Health Organization, 2009; Combs and McClung, 2022). Supplement of PVA in staples such as maize can significantly reduce VAD among under 5 children and pregnant mothers. Biofortification of maize with PVA is thus a rational approach to reduce micronutrient deficiencies for populations that do not have easy access to balanced, diverse diets. Therefore, the HarvestPlus challenges programs that support the development of vitamin A-biofortified maize through conventional breeding in partnership with CIMMYT, IITA, and EMBRAPA (Pixley et al., 2013; Tanumihardjo et al., 2017). Over 50 PVA open-pollinated maize varieties, synthetics, single-cross hybrids, top cross hybrids, and three-way cross hybrids that are high yielding, stress tolerant, profitable, and acceptable to consumers have been released and are contributing to reduced VAD across SSA (HarvestPlus, 2014; Bouis and Saltzman, 2017).

Efforts to improve the nutritional and food security of maize should be however combined with mycotoxin reduction to maximize the health benefits derived from maize. Aflatoxins are highly toxic secondary metabolites produced by several species of *Aspergillus* section Flavi, including *Aspergillus flavus*, *Aspergillus parasiticus*, *Aspergillus parvisclerotigenus*, and *Aspergillus minisclerotigenes* (Amaike and Keller, 2011). There are four major aflatoxins: aflatoxin B1 (AFB1), B2 (AFB2), G1 (AFG1), and G2 (AFG2). The most common and dangerous is AFB1, which is classified as Group 1 carcinogen by the International Agency for Research on Cancer (JECFA, 2018). Chronic and acute aflatoxin exposure have severe repercussions such as impaired food conversion, stunted growth, immune system suppression, hepatocellular carcinoma, and, sometimes, rapid death (International Agency for Research on Cancer, 2002; Williams et al., 2004; Amaike and Keller, 2011). Outbreaks of acute aflatoxicosis have been reported in Kenya and Tanzania (Ochieng et al., 2013; Wild et al., 2015). To prevent aflatoxin exposure, the European Union, the Codex Alimentarius Commission (CAC), and the United States Food and Drug Administration (FDA) established strict regulations for aflatoxins in food and feed and set maximum levels of aflatoxin at 4, 10, and 20 μg/g, respectively (CAC, 2014).

Breeding programs across the globe have made significant investments to identify and release maize germplasm resistant to aflatoxin (Scott and Zummo, 1990; McMillian et al., 1993; Williams and Windham, 2001; Menkir et al., 2008). However, the development of hybrids with stable resistance to aflatoxin is difficult due to the quantitative nature of inheritance of the trait, low heritability, and large genotype by environment interaction effects (Clements et al., 2004; Brooks et al., 2005). In a search for alternative breeding strategies to reduce aflatoxin contamination in maize, breeding for improved PVA content has been a focal point to improve both the quality and safety of human diets (Norton, 1997; Picot et al., 2013; Díaz-Gómez et al., 2016).

Montibus et al. (2015) reported that carotenoids, especially β-carotene (BC) and β-cryptoxanthin (BCX), contribute to the downregulation of aflatoxin biosynthesis by quenching some of the reactive oxygen species (ROS) produced in response to *A. flavus* invasion. The antifungal activities of PVA carotenoids against *A. flavus* and *Fusarium* spp. growth, as well as the inhibition of aflatoxin and fumonisin biosynthesis, have been studied *in vitro* and in the field (Picot et al., 2013; Atanasova-Penichon et al., 2016). Initial experiments by Norton (1997) and Wicklow et al. (1998) suggested that yellow dent maize with high PVA carotenoid content could contribute to reduced aflatoxin accumulation in grain and that the sensitivity of *A. flavus* to carotenoids could be used for assessing aflatoxin resistance in maize. Evaluation of biofortified transgenic maize lines in field trials demonstrated low levels of fumonisin in high carotenoid lines than in their isogenic lines (Díaz-Gómez et al., 2016). Suwarno et al. (2019) assessed aflatoxin accumulation in PVA maize hybrids and reported that hybrids with high concentrations of BC, BCX, and total PVA allowed the accumulation of less aflatoxin than hybrids with lower carotenoid content.

Given that PVA biofortified maize can have an additional health benefit by reducing exposure to aflatoxin, it is possible to combat both VAD and aflatoxin by selecting maize germplasm with positive general combining ability (GCA) effects for PVA carotenoids and negative GCA effects for aflatoxin accumulation. Although published studies reported the correlation of carotenoid content with aflatoxin or fumonisin accumulation (Díaz-Gómez et al., 2016; Suwarno et al., 2019), there is no published report about the potential roles of testers with contrasting PVA content in identifying inbreds having high PVA and supporting reduced fungal infection with subsequent less aflatoxin production.

The objectives of the present study were therefore to i) evaluate aflatoxin resistance among PVA biofortified maize germplasm at IITA, ii) investigate the impact of the suitable tester’s on identifying superior PVA inbreds accumulating high PVA content and less aflatoxin, and iii) identify parental lines of maize combining ability effects for PVA, reduced spores’ production, and reduced aflatoxin accumulation in grain. Our results provide valuable information for breeding programs aiming at developing crops with improved nutritional content and resistance to one of the most dangerous compounds found in nature.

## 2 Materials and methods

### 2.1 Genetic materials and field generation of testcrosses

Sixty PVA maize inbreds with varying levels of PVA (5.4 to 51.7 µg/g) were crossed with two inbred testers having contrasting PVA content (low and high PVA, 14.4 and 25.0 µg/g, respectively) (Menkir et al., 2004; Menkir et al., 2015; Maazou et al., 2022) (**Supplementary Table 1**). The resulting 120 PVA maize hybrids were generated using a line by tester mating design, and four checks made up of hybrid (T1×T2) from the cross between the two testers and three commercial hybrids (Oba Super 2, Ife Hybrid-3, and Ife Hybrid-4) were evaluated in four environments: Ikenne (3°42′ E, 6°54′ N, 30 masl) and Saminaka (8°39′ E, 10°34′ N, 760 masl) in Nigeria during two main rainy seasons of 2020 and 2021 (Maazou et al., 2022). The testcrosses were arranged in a 31 × 4 alpha-lattice design with two replications. Each plot comprised a single 5-m row with a space of 0.75 m between rows and 0.25-m distance between plants, resulting in a population density of 53,000 plants/ha. Recommended cultural practices were followed for optimal production at each location. At harvest, self-pollinated ears of five representative plants were harvested in each plot and bulked to form composite grain samples for laboratory analyses.

### 2.2 Analysis of carotenoids

Carotenoids were extracted at IITA’s Food and Nutrition Laboratory using the method of Howe and Tanumihardjo (2006) and quantified using high-performance liquid chromatography (HPLC; Water Corp., Milford, MA, USA). Extraction and quantification methods were described by Maazou et al. (2022). Briefly, a sub-sample of 0.6-g finely ground maize was mixed with 6 ml of ethanol and 0.1% butylated hydroxyl toluene into a 50-ml glass tube, vortexed for 15 s, and incubated at 85°C in a water bath. Then, solutions of 500 µl of 80% KOH, 200 μl of internal standard β-apo-8′-carotenal, and 4 ml of hexane were added accordingly to the different steps of the extraction procedure. After extraction, 50-μl aliquots of each extract (reconstituted in 500 µl 50:50 methanol/dichloromethane) were injected into the HPLC for analyses of lutein (LT), zeaxanthin (ZX), α-carotene (AC), BC, and BCX. A C30 column (4.6 × 250 mm; 3 μm) eluted by a mobile phase using methanol/water (92: 8 v/v) as solvent A and 100% methyl tertiary-butyl ether (MTBE) as solvent B were used at 450-nm absorbance to separate carotenoids. PVA content (µg/g) was calculated as the sum of BC and half of each BCX and AC content.

### 2.3 Inoculum preparation

The isolate of *A. flavus* La3228 was used to inoculate the PVA hybrid kernels. The isolate belongs to the fungal collection of Pathology and Mycotoxin/Aflasafe Unit of IITA and is frequently used in maize resistance studies at IITA due to its well-characterized virulence and consistency in the production of high aflatoxin levels in maize. Conidia of 7-day-old cultures (31°C, dark) grown in 5/2 agar (5% V-8 juice, 2% Bacto-agar, pH 6.0) were suspended in sterile distilled water. Spores were counted using a hemocytometer and diluted to a final concentration of 4 × 10^6^ conidia/ml for use in kernel screening assays (KSAs).

### 2.4 Inoculation of the kernels

After harvesting, 300-g PVA maize kernels of each hybrid and the checks were sampled and stored in the cold room until use in KSA. The KSA was used to quantify aflatoxin accumulation and spore production (hereafter referred to as spores), as previously described (Ortega-Beltran et al., 2014). Kernels were sorted to remove damaged and undersized ones, and 5 g of each entry was sampled. Kernel samples were then placed in sterile 40-ml glass scintillation vials and surface sterilized by immersion in hot water at 80°C. The kernels were then allowed to dry in a sterile biosafety cabinet. Kernels of each rep of each entry were inoculated with 0.5 ml of the 4 × 10^6^ conidia/ml suspension. Vials were then covered with Tyvek membrane and placed randomly in the incubator for 7 days (31°C, dark). To verify kernels’ viability and absence/presence of *Aspergillus*, five kernels of each maize entry were surface sterilized, placed on modified Rose Bengal agar, and incubated for 7 days.

### 2.5 Quantification of aflatoxins and spore production by *A. flavus*

Aflatoxin quantification was performed as previously described (Ortega-Beltran et al., 2014). Seven days after incubation, 25 ml of methanol was added to each vial to stop the fungal growth. The vials were then swirled to dislodge the spores from the kernels, and 1 ml of the suspension was transferred into a sterile Eppendorf tube for spore quantification. The remaining mixture was blended with a laboratory blender (Warring Commercial, Torrington, CT, USA) for aflatoxin quantification. The blended mixture was poured into a sterile 250-ml plastic conical flask and sealed with Parafilm. The blender was decontaminated with 80% ethanol between samples to avoid cross-contamination. The mixture was shaken on an orbital shaker (400 rpm for 30 min) and then filtered into a sterile 150-ml plastic beaker using Whatman filter paper (Whatman Intl. Ltd., Maidstone, UK). The filtrate was transferred into a 50-ml conical flask, and 10 ml of dichloromethane and 5 ml of distilled water were added, shaken gently, and allowed to separate. The extract in the bottom was transferred into a plastic beaker and dried overnight in a fume hood. The extracts were dissolved with 2 ml of dichloromethane and transferred to Eppendorf tubes. Extract measuring 40 µl was directly spotted in duplicate alongside aflatoxin standards on thin-layer chromatography plates (silica gel 60, EMD Millipore, Darmstadt, Germany) and developed with diethyl ether–methanol–water (96:3:1). The plates were examined under UV light for qualitative assessment of aflatoxins (positive = blue fluorescence, negative = no fluorescence). Plates were then subjected to scanning densitometry using a CAMAG TLC Scanner 4 and quantification software VisionCats 3.1 (CAMAG AC, Muttenz, Switzerland).

Spores were quantified using a turbidimeter (Orbeco Analytical Systems Inc., Farmingdale, NY, USA). Spore suspensions washed from infected kernels (1 ml of the 25 ml total) were diluted 20-fold with 50% methanol and measured in nephelometric turbidity units (NTU; *y* = 49,937*x*; *x* = NTU, *y* = spores/ml).

### 2.6 Statistical analysis

Normality of frequency distributions was assessed using SAS v9.4 (SAS Institute Inc, 2012). Total aflatoxin (B1+B2) and spore data were transformed using the common logarithm (Log_10_) for normalization of variances. Data for each location (site–year) was analyzed separately using a linear model with hybrids, lines, and testers considered as fixed effects. Given the similar observed trends in the two locations (**Supplementary Table 2**), we decided to compute the combined analysis of variance (ANOVA) following the line × tester procedure as suggested by Singh and Chaudhary (1977) using SAS Proc mixed procedure. In the combined analysis, each location–year combination was considered an environment. Environment, replication (Env), block (replication × Env), and Env × hybrid interactions were considered as random effects, while hybrid, lines, and testers, were considered as fixed effects in the linear model.

The efficiency of testers was first determined based on the genetic variance estimates obtained from the combined ANOVA of testcrosses means of each tester across the four Env (Castellanos et al., 1998). Then, the proportional contributions of line, tester, and line × tester were estimated. The genotypic correlations of aflatoxin and spores with carotenoids were estimated using the Proc corr procedure in SAS as well as the regression between PVA and aflatoxin content using Proc Reg.

Standard heterosis (H) was calculated for each testcross using the heterosis equation described by Fan et al. (2016): H = 100% × (F_1_ − CK)/CK, where F_1_ is the aflatoxin accumulated in a testcross and CK is the aflatoxin accumulation in the hybrid generated by cross of the two testers (T1 × T2).

The GCA and specific combining ability (SCA) effects of the inbreds and testers and the variance components for aflatoxin, spores, and PVA were calculated using the analysis of genetic design software (AGD-R, V.5.0; Rodríguez et al., 2018). The relative importance of GCA and SCA effects for each trait was estimated using the general predicted Baker ratio: GCA/SCA = 2 MSGCA/(2MSGCA + MSSCA), where MSGCA = mean square for GCA and MSSCA = mean square for SCA. A ratio of >0.5 implies that GCA is more important than SCA in the inheritance of the character, while a ratio of<0.5 implies that SCA is more important than GCA (Baker, 1978). Hybrids were classified using K-means to group them as tolerant (T), moderately tolerant (MT), and susceptible (S). The BLUES values were used to perform the non-hierarchical K-means cluster analysis using the factoextra v1.0.7 package of R software v4.1.0. Eta-squared (η^2^ = SSeffect/SStotal) was used to determine the proportion of variation according to Cohen’s guidelines (≤0.01 = small, ≤0.06 = medium, and ≥0.14 = large effect size) using lsr package v0.5.2.

## 3 Results

### 3.1 Combined analysis of variance

The combined ANOVA showed that environment (Env) had a significant effect (*p*-range:<0.01 to<0.0001) on aflatoxin accumulation, spores, PVA, BC, BCX, AC, LT, and ZX. The GCA effects among the 60 inbreds and the two testers were significant (*p* < 0.01–0.001) for all traits (**Table 1**). The variations among the 120 hybrids were also significant for all the traits (*p* < 0.01–0.001). Further, interactions of Hybrid × Env, Line × Env, Tester × Env, and Line × Tester (SCA) were significant (*p* < 0.05–0.0001) for most of the traits measured. The mean square for testers was much higher than that of the lines, indicating that the tester effects had a greater impact than the line effects. Tester × Env interaction was much higher than the Line × Env interaction. Line × Tester and Line × Tester × Env interactions were not significant for spores, indicating that the quantity of spores is less predictable to quantify aflatoxin. The repeatability for log-transformed aflatoxin values and carotenoids ranged from moderate to high (0.59 to 0.95) but was low for spores (0.21). Baker’s ratios of GCA/SCA effects were >0.5 for aflatoxin, spores, and carotenoids, varying from 0.96 to 0.99, suggesting the predominance of additive over non-additive gene effects (**Table 1**).

**Table 1:** Mean squares from the combined analysis of variance for spores, aflatoxin accumulation, and content of provitamin A (PVA) of maize kernels of 60 PVA maize inbreds crossed to two testers containing contrasting levels of PVA and others evaluated in four environments in Nigeria.

Source of variation	DF	Aflatoxin	No. of spores	Lutein	Zeaxanthin	β-Cryptoxanthin	α-Carotene	β-Carotene	Provitamin A
**Env**	3	15.69†	4.06†	722.67**	385.65**	12.51**	2.57**	829.52**	909.02**
**REP (Env)**	4	0.17	0.01	77.73**	93.37**	9.3**	1.05**	34.71**	57.67**
**Block (Env × Rep)**	240	0.18	0.06*	4.64**	5.88**	0.4**	0.08**	2.48**	3.04**
**Hybrid (H)**	123	0.55†	0.08†	28.6**	61.85**	8.35**	0.18**	34.27**	25.83**
**Hybrid × Env**	369	0.24**	0.07†	3.45**	3.31	0.37*	0.06**	3.15**	3.25**
**Line (GCA)**	59	0.60†	0.10†	75.13**	85.35**	15.26**	0.34**	50.32**	45.35**
**Tester (GCA)**	1	17.00†	1.51†	367.23**	5,215.77**	388.35**	2.58**	2,438.68**	1,498.54**
**Line × Tester (SCA)**	59	0.37†	0.06	15.11**	10.11**	1.63**	0.1**	6.22**	5.94**
**Line × Env**	177	0.24**	0.07**	4.41*	5.22	0.53	0.09**	4.97**	5.17**
**Tester × Env**	3	0.91**	1.10†	66.89**	42.52**	7.39**	0.73**	67.63**	85.09**
**Line × Tester × Env**	177	0.26***	0.06	4.71*	4.54	0.47*	0.07	3.07**	3.48**
**Error**	252	0.18	0.04	2.11	3.03	0.29	0.04	1.29	1.37
**Repeatability**		0.59	0.21	0.89	0.94	0.95	0.65	0.91	0.87
**CV (%)**		8.92	9.22	18.41	17.94	13.6	24.42	12.64	10.25
**Ratio GCA/SCA**		0.97	0.96	0.98	0.99	0.99	0.98	0.99	0.99

*, **, ***, and † indicate significance at probability < 0.05, 0.01, 0.001, and 0.0001 levels, respectively.

GCA, general combining ability; SCA, combining ability.

The contribution of Line, Tester, and Line × Tester to the total variance were 3.3%, 94.6%, and 2.1%, respectively, for spore; 5.9%, 90.7%, and 3.4%, respectively, for aflatoxin; and 2.9%, 96.7%, and 0.4%, respectively, for PVA content. The genetic variances estimated for the testcrosses involving T2 with low PVA content were slightly higher for aflatoxin, spores, PVA, and most other carotenoids (**Table 2**). The genetic variances for T1 with high PVA were the highest only for BCX while displaying relatively similar genetic variance with T2 for other traits.

**Table 2:** Genetic variance among testcrosses with two testers with contrasting provitamin A content (T1 and T2) under *Aspergillus flavus* inoculation.

Traits	T1	T2
**Aflatoxin**	1.57 ± 0.06	2.57 ± 0.06
**No. of spores**	1.05 ± 0.02	1.25 ± 0.03
**Lutein**	9.61 ± 0.67	10.58 ± 0.55
**Zeaxanthin**	9.71 ± 0.56	9.81 ± 0.68
**β-Cryptoxanthin**	16.97 ± 0.25	16.55 ± 0.27
**α-Carotene**	2.47 ± 0.04	2.69 ± 0.05
**β-Carotene**	6.58 ± 0.57	8.29 ± 0.38
**Provitamin A**	5.25 ± 0.51	7.49 ± 0.40

### 3.2 Spore count and aflatoxin contamination of the PVA hybrids

**Figure 1:**
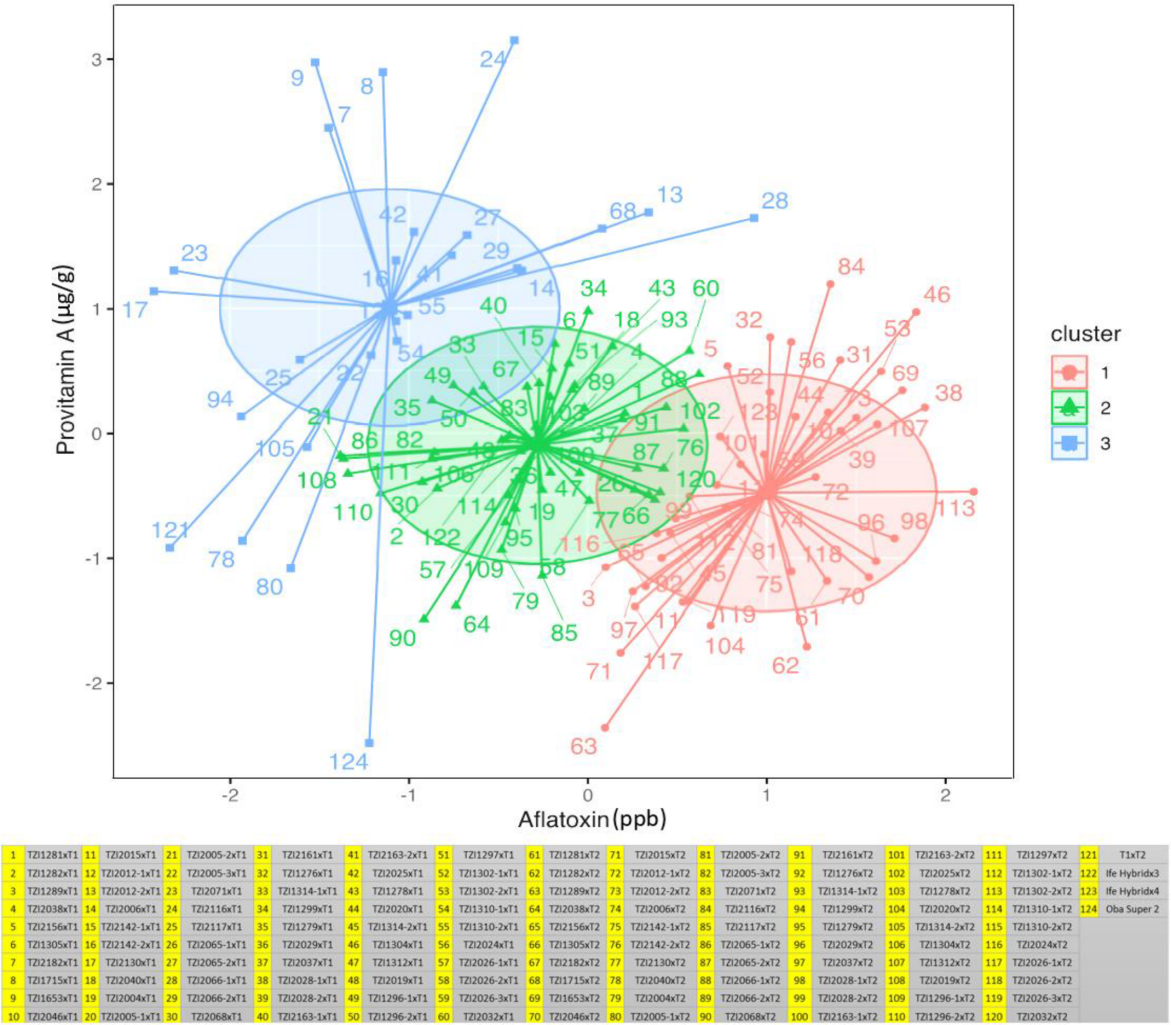
Testcrosses grouping maize germplasm as susceptible (cluster 1), moderately tolerant (cluster 2), and tolerant (cluster 3) categories based on their aflatoxin and provitamin A content.

In the combined ANOVA, significant differences (*p* < 0.01–0.001) were found among hybrids for aflatoxin, spore count, and PVA content. Testcross means varied from 3.9 to 5.5 ppb for aflatoxin, 1.93 to 2.57 NTU for spore count, and 6.2 to 18.4 µg/g for PVA content (**Supplementary Table 3**). The result of the K-means analysis for aflatoxin classified 30 testcrosses as tolerant, 34 as moderately susceptible, and 56 as susceptible (**Figure 1**; **Supplementary Table 3**). Among tolerant testcrosses, 63.3% were crossed with T1, and the remaining 36.7% were crossed with T2. Among testcrosses classified as susceptible, 37.5% involved T1, while 62.5% involved T2. Among the moderately tolerant testcrosses, 58.8% were crossed to T1, whereas 41.2% were crossed with T2. With the use of spore counts, 45 testcrosses were classified as tolerant with 48.9% crossed with T1 and 51.1% with T2, 45 testcrosses were moderately tolerant with 51.1% crossed with T1 and 48.9% with T2, and 30 testcrosses were found to be susceptible with 50% each crossed with T1 and T2. The most resistant testcrosses in terms of spore production and aflatoxin accumulation were TZI2130, TZI2071, TZI2117, TZI1653, TZI2182, TZI2005-2, TZI2005-3, and TZI1715 involving T1, and TZI1314-2, TZI2005-1, TZI1299, TZI2040, TZI2065-1, TZI2019, and TZI1296-2 involving T2. The check Oba Super 2 was classified as tolerant based on both aflatoxin and spore count, Ife Hybrid-3 was moderately tolerant and tolerant, respectively, and Ife Hybrid-4 was susceptible and tolerant, respectively. T1×T2 hybrid was tolerant to both aflatoxin and spore count.

As per the standard HarvestPlus classification, 45 testcrosses had high PVA content (12 to 18.4 µg/g) with 82.2% of them being crosses of T1. Seventy-one testcrosses had medium PVA content (8.1 to 11.9 µg/g) of which 31% were crosses of T1 and 69% were crosses of T2. Four testcrosses having low PVA levels were crosses of T2 (**Supplementary Table 3**). Among the 120 testcrosses, 61 had higher PVA content (12.1 to 18.4 µg/g) than the best-performing commercial hybrid check Ife Hybrid-4 (11.4 µg/g). Interestingly, among the testcrosses classified as tolerant to aflatoxin, 16 had higher PVA content, 15 had medium PVA levels, and none had low PVA content (**Figure 1**; **Table 3**; **Supplementary Table 3**). However, among the 45 high PVA testcrosses, 31 were classified as tolerant to moderately tolerant to aflatoxin, while 14 were classified as susceptible. The 10 best testcrosses combining high PVA content with tolerance to aflatoxin were TZI1653×T1, TZI1715×T1, TZI2182×T1, TZI2025×T1, TZI2065-2×T1, TZI2163-2×T1, TZI2142-2×T1, TZI2071×T1, TZI2130×T1, and TZI1310-2×T1 (**Table 3**; **Supplementary Table 3**).

**Table 3:** Performance of the 10 best and bottom combinations of inbreds and testers (T1 and T2) for tolerance to spores and aflatoxin accumulation, desirable GCA, SCA, and heterosis for aflatoxin accumulation and PVA content. .

Mean performance				GCA					SCA		Heterosis	
Line	Tester	AFLA	NTU	PVA	AFLA	NTU	LT	BC	PVA	AFLA	PVA	AFLA	PVA
**TZI2071**	T1	3.978T	2.18MT	14.33H	−0.49†	−0.2**	2.0***	2.1***	1.46**	−0.17	0.13	−11.8	52.4
	T2	5.17MT	2.02T	11.55M						0.21	−0.13	14.5	22.9
**TZI1715**	T1	4.57T	2.22MT	17.85H	−0.27*	−0.09	−2.98†	5.35†	4.99†	0.33*	0.14	1.4	89.9
	T2	4.44MT	2.12T	15.07H						−0.3*	−0.14	−1.6	60.3
**TZI2066-2**	T1	3.95MT	2.00T	14.36H	−0.19	−0.08	−0.18	2.14***	1.63**	−0.31*	0.00	−12.5	52.7
	T2	4.98MT	2.25MT	11.86M						0.31*	0.00	10.5	26.2
**TZI2065-2**	T1	4.64T	2.24T	14.95H	−0.14	−0.12	−0.87	2.08***	1.33*	0.08	0.82	2.9	59.0
	T2	5.03S	2.21MT	10.79M						−0.08	−0.82	11.6	14.8
**TZI2163-1**	T1	4.83MT	2.49T	12.31H	−0.02	−0.09	−1.37**	1.49**	1.07	0.09	−0.45	7.0	30.9
	T2	4.58MT	2.00T	10.71M						−0.09	0.45	1.4	13.9
**TZI2142-1**	T1	4.40MT	2.47S	12.57H	−0.12	0.01	−1.5**	1.52**	1.17*	−0.09	0.13	−2.4	33.8
	T2	4.98S	2.29MT	9.81M						0.09	−0.13	10.5	4.4
**TZI1653**	T1	4.65T	2.34T	18.03H	−0.12	0.01	4.82†	3.55†	3.63†	−0.06	1.7***	3.0	91.8
	T2	4.98S	2.29MT	12.18H						0.06	−1.7***	10.5	29.6
**TZI2182**	T1	4.00T	2.25T	16.86H	−0.16	0.04	−2.47†	3.86†	3.04†	−0.17	1.02*	−11.2	79.4
	T2	5.12MT	2.27S	12.25H						0.17	−1.02*	13.4	30.3
**TZI2006**	T1	4.76MT	2.17MT	14.32H	−0.07	0.003	−1.46**	1.04	0.77	0.02	0.81	5.6	52.3
	T2	4.47S	2.23MT	10.18M						−0.02	−0.81	−1.0	8.3
**TZI1278**	T1	4.40S	2.19T	12.98H	−0.15	−0.15*	−1.21*	0.55	1.04	0.09	−0.79	−2.4	38.1
	T2	4.81MT	2.06T	12.06H						−0.08	0.79	6.7	28.3
**TZI2068**	T1	4.16T	2.19T	10.56M	−0.53†	−0.12	1.51**	−1.33*	−2.1***	0.02	−0.03	−7.8	12.3
	T2	4.43T	2.29T	8.12M						−0.03	0.03	−1.8	−13.6
**TZI2117**	T1	4.02T	2.19T	12.74H	−0.4***	−0.01	5.62†	0.32	−0.65	−0.06	0.66	−10.8	35.5
	T2	4.57MT	2.19T	8.90M						0.06	−0.66	1.3	−5.3
**TZI2019**	T1	4.71MT	2.19T1	11.38M	−0.28*	−0.02	0.37	−0.68	−0.42	0.23	−0.91*	4.4	21.0
	T2	4.37T	2.08MT	10.71M						−0.23	0.91*	−3.2	13.9
**TZI2005-1**	T1	4.47MT	2.1T	11.53M	−0.18	−0.04	1.14*	−1.31*	−1.18*	0.07	0.00	−0.8	22.7
	T2	5.06T	2.22MT	9.03M						−0.07	0.00	12.2	−3.9
**TZI2046**	T1	5.13S	2.19S	11.71M	0.51†	0.02	−2.32†	−1.82**	−1.18*	0.00	0.17	13.8	24.6
	T2	5.55S	2.24T	8.86M						0.00	−0.17	23.1	−5.7
**TZI2028-2**	T1	5.19S	2.19S	11.47M	0.29*	0.03	1.03*	−1.07	−0.82	0.24	−0.51	15.0	22.0
	T2	4.71S	2.19S	9.91M						−0.24	0.51	4.4	5.4
**TZI2028-1**	T1	4.67S	2.19MT	11.86M	0.24*	0.09	0.47	−0.97	−0.75	−0.07	−0.08	3.5	26.3
	T2	5.48S	2.35MT	9.56M						0.07	0.08	21.6	1.6
**TZI2012-2**	T1	4.96S	2.19MT	15.36H	0.28*	0.01	−1.11*	0.26	0.55	0.14	0.58	9.9	63.4
	T2	4.96S	2.46S	11.69M						−0.14	−0.58	10.0	24.4
**TZI2026-3**	T1	4.68S	2.19T	11.04M	0.09	−0.12	−0.51	−1.7**	−1.73**	0.00	0.05	3.7	17.4
	T2	5.18S	2.29T	8.44M						0.00	−0.05	14.8	−10.3
**TZI2020**	T1	4.90S	2.19T	11.78M	0.21	−0.002	1.9***	−1.9***	−1.57**	0.23	0.63	8.6	25.4
	T2	5.12S	2.24T	8.01L						−0.23	−0.63	13.5	−14.8

*, **, ***, and † indicate significance at probability< 0.05, 0.01, 0.001, and 0.0001 levels, respectively.

Afla, Aflatoxin, log10 transformed sum of the average total aflatoxin (B1 and B2) produced by Aspergillus flavus La3228.

NTU, log10 transformed of the nephelometric turbidity unit, 1 NTU = 49,937 spores. K-means classification: T, tolerant to aflatoxin, spores; MT, moderately tolerant; S, susceptible; H, high provitamin A; M, medium; L, low; GCA, general combining ability; SCA, combining ability; PVA, provitamin A.

**Figure 2:**
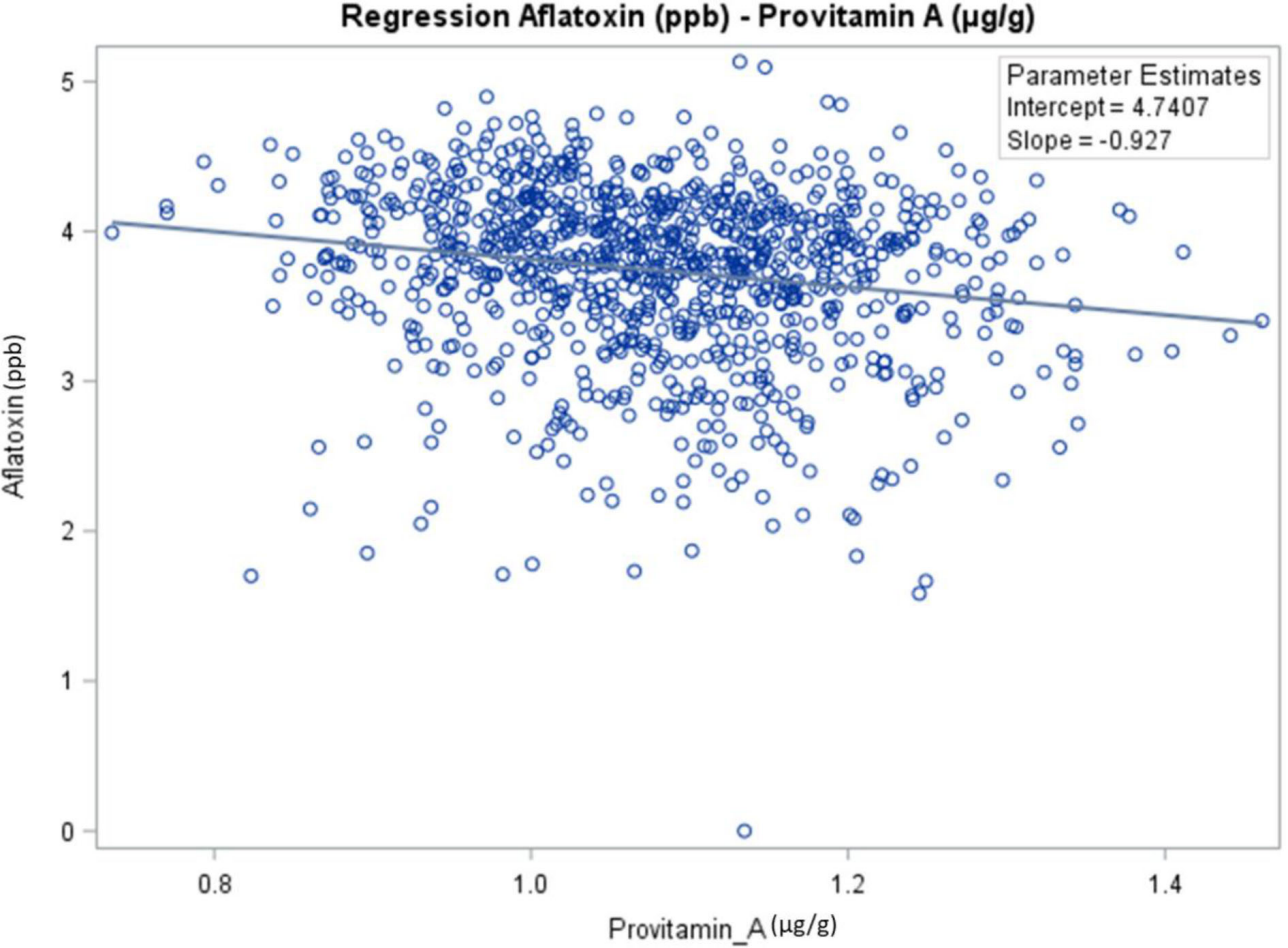
Regression analysis to determine the relationship between aflatoxin contamination and content of provitamin A in maize hybrids evaluated in the current study.


The genetic correlation showed that aflatoxin was negatively correlated with BC (*r* = −0.29, *p* < 0.0001) and PVA (*r* = −0.23, *p* < 0.0001) (**Table 4**). Aflatoxin was positively correlated with spore count (*r* = 0.24, *p* < 0.0001), LT (*r* = 0.06, *p* < 0.05), ZX (*r* = 0.23, *p* < 0.0001), BCX (*r* = 0.20, *p* < 0.0001), and AC (*r* = 0.24, *p* < 0.0001). As seen on the slope of the regression (**Figure 2**), the PVA content has a significant linear effect on aflatoxin. Estimates obtained from the regression equation are the following:

**Table 4:** Genotypic correlations among aflatoxin content, spore count, provitamin A (PVA), and other carotenoids of 120 PVA maize testcrosses.

	Aflatoxin	No. of spores	Lutein	Zeaxanthin	β-Cryptoxanthin	α-Carotene	β-Carotene	Provitamin A
**Aflatoxin**	1	0.24†	0.06*	0.23†	0.20†	0.23†	−0.29†	−0.23†
**No. of spores**		1	0.06	−0.11***	0.05	0.08*	0.20†	0.22†
**Lutein**			1	0.14†	−0.11***	0.13†	−0.01	−0.04
**Zeaxanthin**				1	0.62†	0.38†	−0.44†	−0.3†
**β-Cryptoxanthin**					1	0.54†	−0.18†	0.06
**α-Carotene**						1	−0.03	0.15†
**β-Carotene**							1	0.96†
**Provitamin A**								1

*, **, ***, and † indicate significance at probability< 0.05, 0.01, 0.001 and 0.0001 levels, respectively.

$${\text{Log}}_{10}\text{Aflatoxin}=4.74-0.93\log_{10}\text{PVA};\left(\text{t}-\text{value}=-5.68,p<0.001\right)$$

Impacts of aflatoxin, spores, and PVA to the testcrosses on aflatoxin resistance determined with the eta-squared showed that the PVA effect (η^2^ = 0.36) was larger than spore count (η^2^ = 0.13), indicating that PVA had a more significant effect on tolerance and susceptibility of the hybrids to aflatoxin.

The percentage of heterosis over the check (T1×T2) for aflatoxin, spores, and PVA is presented in **Supplementary Table 4**. The standard heterosis ranged from −12.5% to 23.1% for aflatoxin, −20.0% to 6.1% for spores, and −34.1% to 95.9% for PVA. Among the testcrosses, 26 had negative heterosis for aflatoxin (range = −12.5% to −0.8%). Among these, 21 testcrosses involved T1 with aflatoxin heterosis ranging from −12.5% to −0.8%, and five testcrosses involved T2 with −3.3% to −1.0% standard heterosis. For spores, negative heterosis was observed in 93 testcrosses. Among these, 43 testcrosses involving T1 displayed negative heterosis varying from −17.2% to −0.1%, while 50 testcrosses involving T2 showed −20.0% to −0.6% standard heterosis. For PVA, standard heterosis for 58 testcrosses involving T1 ranged from 9% to 96% and from 1% to 60% for 42 testcrosses involving T2. Interestingly, 18 testcrosses out of the 26 displaying negative standard heterosis for aflatoxin also had negative heterosis for spores and positive heterosis for PVA, while three testcrosses—TZI1289, TZI2142-1, and TZI1312—involving T1 displayed negative heterosis for aflatoxin (−10.8%, −2.4%, and −1.1%, respectively) and positive for spores (5.5%, 2.1%, and 6.1%, respectively) and PVA (35.5%, 33.8%, and 14.0%, respectively).

### 3.3 Estimates of combining ability effects

The estimated GCA of the 60 inbreds and the two testers is presented in **Tables 3**, **5**, and **Supplementary Table 5**. T1 had significant negative GCA for aflatoxin, positive GCA for spores, and significant positive GCA for PVA and other carotenoids but significant negative GCA for ZX and BCX (**Table 5**). In contrast, T2 had significant positive GCA for aflatoxin, negative GCA for spores, and significant and negative GCA effects for PVA, LT, and BC. However, it had a significant positive GCA for ZX and BCX. Twenty-five inbreds had negative GCA effects for aflatoxin, with six of them (TZI2071, TZI1715, TZI2068, TZI2117, TZI2019, and TZI2004) having significant negative GCA for aflatoxin (**Table 3**; **Supplementary Table 5**). Among the 25 inbreds, 15 showed negative GCA for spores with TZI2071 and TZI1278 having significant negative GCA. Only two inbreds, TZI2071 and TZI1715, there was combined significant negative GCA for aflatoxin and spores with significant positive GCA for BC and PVA. Four inbreds (TZI2046, TZI2028-2, TZI2026-3, and TZI2020) had positive to significant positive GCA for aflatoxin and negative to significant negative GCA effects for PVA (**Supplementary Table 5**).

**Table 5:** Estimation of the general combining ability (GCA) effects for the two testers evaluated across four environments.

Traits	T1	T2
**Aflatoxin**	−0.13**	0.13
**No. of spores**	0.04	0.04
**Lutein**	0.62*	−0.62*
**Zeaxanthin**	−2.33**	2.33**
**β-Cryptoxanthin**	−0.64**	0.64**
**α-Carotene**	−0.05	0.05
**β-Carotene**	1.59**	−1.59**
**Provitamin A**	1.25**	−1.25**

* and ** indicate significance at probability< 0.05 and 0.01 levels, respectively.

Inbreds TZI2068, TZI2117, and TZI2019 had significant negative GCA effects for both aflatoxin and PVA and significant positive GCA for LT (**Table 3**). Similarly, inbreds TZI2005-3, TZI2038, TZI2015, TZI2040, and TZI2005-1 had significant negative GCA for both aflatoxin and PVA and significant positive GCA for LT.

Among the 120 hybrids, 57 had negative SCA effects for aflatoxin. Among those, seven testcrosses (TZI1312×T1, TZI1314-2×T2, TZI2066-2×T1, TZI1715×T2, TZI2015×T1, TZI2024×T2, and TZI2032×T2) had significant negative SCA for aflatoxin and spores (**Supplementary Table 6**). These hybrids had negative SCA for PVA, except TZI2066-2×T1, showing positive SCA effects but non-significant. Among the testcrosses combining negative SCA for aflatoxin with positive SCA for PVA, 20 testcrosses involved T1, and 15 involved T2. The testcrosses TZI1312×T2, TZI1715×T1, TZI2066-2×T2, TZI2015×T2, TZI2024×T1, and TZI2032×T1 had significant positive SCA for aflatoxin, positive SCA for spores (except TZI2024×T1), and PVA, with TZI1312×T2 having significant positive SCA for PVA.

None of the testcrosses combined significant negative SCA for aflatoxin and spores with significant positive SCA for PVA. However, the testcrosses involving inbreds TZI2182 and TZI1653 with T1 and TZI2019 with T2 had negative SCA effects for aflatoxin and spores and significant positive SCA for BC and PVA (**Table 3**; **Supplementary Table 6**). Similarly, the testcross involving TZI2066-2 with T1 showed significant negative SCA for aflatoxin, negative SCA for spores, and positive SCA for PVA. In total, 31 testcrosses had negative SCA for aflatoxin and positive SCA for PVA.

## 4 Discussion

We evaluated PVA biofortified maize testcrosses developed at IITA for resistance to aflatoxin accumulation and spore production by *A. flavus*. We detected high variability among PVA inbreds and their testcrosses in susceptibilities to spore production and subsequent aflatoxin contamination. Several inbreds and testcrosses having the potential to contribute to aflatoxin resistance while harboring high PVA content were detected. Large-scale use of biofortified PVA maize to alleviate VAD to maize consumers, particularly in SSA, may provide additional benefits of reducing aflatoxin exposure in the populations. The carotenoid content of PVA maize has been reported to contribute to aflatoxin resistance (Norton, 1997; Wicklow et al., 1998; Díaz-Gómez et al., 2016; Suwarno et al., 2019).

Indeed, it has been reported that PVA maize developed for cultivation in SSA harbors resistance to aflatoxin (Suwarno et al., 2019). Therefore, our results hold promise for the development of tropical germplasm with effective resistance to aflatoxin and high PVA content. Our studies were based on a rapid laboratory-based KSA, which is useful to study resistance to aflatoxin production in maize. The KSA has been used to develop aflatoxin-resistant maize inbreds (Menkir et al., 2006; Menkir et al., 2008).

The germplasm evaluated in the current study was grown in two locations in Nigeria, Ikenne and Saminaka, for 2 years. Results were combined per location because similar trends were detected within locations. However, there were some cases in which variation in aflatoxin accumulation among replications occurred, which may be partly attributed to edaphic factors. Similar results were reported by Suwarno et al. (2019), who found a large variation of aflatoxin accumulation among five environments and even among micro-environments (replications) when evaluating aflatoxin resistance of 144 F_1_ PVA hybrids inoculated with *A. flavus*. Large environmental influences on aflatoxin accumulation during both pre- and post-harvest have been reported (Mayfield et al., 2011; Warburton et al., 2011). It is therefore required to evaluate germplasm in multiple locations over multiple years to further validate the role of PVA maize in reducing aflatoxin accumulation.

Resistance proteins expressed in the kernel may reduce fungal growth and aflatoxin accumulation. Intuitively, reduced fungal growth may be perceived as a sign of resistance to aflatoxin. In our study, the genotypic correlation between spore production and aflatoxin accumulation was significant and positive, agreeing with the results of others. Brown et al. (1995) reported that resistance to aflatoxin production is directly related to resistance to fungal colonization in certain genotypes. Further, Luna-López et al. (2013) tested the resistance of 11 genotypes of immature maize grain to two toxigenic strains of *A. flavus* and reported a significant association between fungal growth and aflatoxin content. In our study, however, there were some testcrosses such as TZI1282×T1, TZI2065-1×T2, and TZI2005-3×T1 that accumulated less aflatoxin but allowed higher production of spores (**Supplementary Table 3**). Similarly, testcrosses of TZI2026-3 and TZI2020 with the two testers were susceptible to aflatoxin contamination but allowed the production of fewer spores (**Table 3**). These contrasting results tend to suggest ways of detecting the presence of aflatoxin in maize. Brown et al. (2001) reported susceptibility to fungal growth in a maize line that accumulated low levels of aflatoxin and conversely an aflatoxin-susceptible inbred supporting low levels of fungal growth. However, reports of positive or negative correlation coefficients between spore colonization and aflatoxin contamination demonstrate there are no definitive relationships between these two traits (Brown et al., 1999; Luna-López et al., 2013; Ortega-Beltran et al., 2014). Therefore, resistance to aflatoxin accumulation is most reliably detected by direct aflatoxin quantification (Brown et al., 1999; Ortega-Beltran et al., 2014). We calculated eta-squared values for PVA and spores to examine their usefulness in predicting resistance to aflatoxin. Eta-squared values for PVA were larger than those of spores, indicating that spore values were less reliable predictors of resistance to aflatoxin. Thus, PVA content appears to be more important in predicting aflatoxin resistance.

Earlier studies highlighted the potential involvement of antioxidant compounds such as carotenoids (α-tocopherol, LT, ZX, BCA, AC, BC, and ferulic acid) to quench oxygen free radicals produced by plant cells as a defense response, contributing to reduced oxidative stress that modulates aflatoxin and fumonisin biosynthesis (Norton, 1997; Picot et al., 2013; Díaz-Gómez et al., 2016). Abscisic acid hormone derived from the enzymatic oxidation of carotenoids is involved in responses to environmental stresses and pathogen attacks (Hakeem et al., 2014). Montibus et al. (2015) have reported that carotenoid content contributes to the downregulation of aflatoxin biosynthesis. Therefore, PVA carotenoids can have important signaling functions in ROS, reducing *A. flavus* infection and subsequently aflatoxin accumulation. In the current study, testcrosses classified tolerant to aflatoxin (n = 30) had PVA content varying from medium to high (8.1 to 18.0 µg/g). In contrast, susceptible testcrosses (n = 56) had low to medium PVA content (5.6 to 8.0 µg/g). Only BC and PVA were significantly and negatively correlated with aflatoxin accumulation, consistent with the results of Suwarno et al. (2019). However, the magnitude of the relationship between aflatoxin and each of BC and PVA in our study was small (*r* = −0.29, *p* < 0.0001 for BC; *r* = −0.23, *p* < 0.0001 for PVA), suggesting that further research is needed to confirm the findings. Noteworthy is that the commercial hybrid check Oba Super 2, with the lowest PVA content, accumulated lower aflatoxin as 13 testcrosses combining low aflatoxin and high PVA content. The mechanisms of resistance in Oba Super 2 thus deserve further investigation to determine if it is dependent on protein expression, wax and/or cutin content, or kernel architecture.

Significant research efforts have allowed releasing germplasm resistant to aflatoxin (Scott and Zummo, 1990; McMillian et al., 1993; Williams and Windham, 2001; Menkir et al., 2008). In this process, it is essential to select appropriate testers that correctly classify the combining abilities (GCA and SCA) of inbreds for generating hybrids with resistance to aflatoxin production and high PVA content (Guimarães et al., 2012). The significant GCA effects of both inbreds and testers for aflatoxin, spores, PVA, and other carotenoids as well as the significant SCA effects for these traits imply that both additive and non-additive gene effects were important in controlling those traits. Moreover, the high GCA/SCA ratio value for all the traits except for spores is an indicator of additive gene action being more important than non-additive gene action in controlling the inheritance of these traits. These results concur with those of Suwarno et al. (2019), who assessed the combining ability, aflatoxin, and carotenoid content of maize inbreds in hybrid combinations. Other studies also reported that additive gene action was more important than non-additive gene action in the inheritance of aflatoxin resistance in maize (Warburton and Williams, 2014; Meseka et al., 2018).

Among the eight superior inbreds, TZI2071 and TZI1715 are the most promising parental materials because they combine significant negative GCA effects for aflatoxin and spores with significant positive GCA effects for PVA (**Table 3**). These inbreds can be used to improve PVA content and reduce aflatoxin accumulation. Inbreds TZI2065-2 and TZI2142-1 had positive GCA for grain yield (Maazou et al., 2022), and we found them to have desirable GCA for aflatoxin, spores, and PVA.

We found testcrosses (**Supplementary Table 3**) involving TZI2182 and TZI1653 containing PVA exceeding the HarvestPlus target of 15 µg/g that also accumulated low aflatoxin. This could arise from the negative GCA effects for aflatoxin and the significant positive GCA effects for PVA of TZI2182 and TZI1653. The high PVA tester (T1), which identified these inbreds, could be considered a suitable tester. PVA inbreds had greater heterosis for resistance to aflatoxin when crossed with T1, and 14 of the 18 testcrosses showed negative standard heterosis for aflatoxin and spores, and positive heterosis for PVA involved T1. As a result, these inbreds can be used for developing high PVA hybrids with resistance to aflatoxin.

The GCA effects of testers were greater than those of inbreds in conferring high PVA, reduced spores, and aflatoxin accumulation. T2 had slightly higher genetic variances for aflatoxin, spores, PVA, and other carotenoids, except for BC, whereas the genetic variances for T1 were higher only for BCX (**Table 2**). Thus, T2 can be used to discriminate inbreds for both PVA content and resistance to aflatoxin. A low-performing tester with a low frequency of favorable alleles at important loci would be more effective in discriminating the potential value of inbreds (Rawlings and Thompson, 1962; Hallauer and Lopez-Perez, 1979). However, testcrosses involving T1 were the most resistant to aflatoxin and had moderate to high PVA content than testcrosses with T2. T1 had significant negative GCA for aflatoxin and significant and positive GCA for LT, BC, and PVA (**Table 5**). The most promising parental lines (TZI2071, TZI1715, TZI2066-2, TZI2065-2, TZI2163-1, TZI2142-1, TZI1653, and TZI2182) forming testcrosses combining negative SCA for aflatoxin with positive SCA for PVA involved T1, which is consistent with the results of Zebire et al. (2020). Therefore, T1 provided a greater opportunity to characterize the PVA inbreds and can then be used as a suitable tester for identifying parental lines to develop high PVA maize hybrids with resistance to aflatoxin. Because breeding for aflatoxin resistance has proven to be difficult due to the quantitative nature of the trait, low heritability, and large genotype by environment interaction effects, identifying and selecting parental lines with high PVA tester to develop high PVA hybrids could be used for reducing aflatoxin contamination in maize.

## 5 Conclusion

We evaluated PVA biofortified maize inbreds developed at IITA to determine the usefulness of testers with contrasting PVA content in identifying inbreds combining high PVA content with resistance to aflatoxin contamination as parents for breeding. Our findings demonstrated that high PVA maize hybrids had reduced aflatoxin levels in their grains, although the relationship between aflatoxin and PVA was not very strong. Further investigations are thus needed to determine whether high PVA has a significant effect on *A. flavus* spores and aflatoxin contamination as well as other mycotoxin producers such as *A. minisclerotigenes*, *A. parasiticus*, and *Fusarium* spp.

The significant GCA and SCA effects of both lines and testers for all the traits imply that both additive and non-additive gene effects were important in conditioning the traits with a prominence of additive gene action (ratio GCA/SCA > 0.5). The high PVA tester T1 had desirable GCA effects for aflatoxin, LT, BC, and PVA and was useful to discriminate the PVA inbreds for PVA content and aflatoxin accumulation. T1 was identified as the most promising parental line that significantly reduced aflatoxin contamination while increasing PVA content. Thus, T1 can be used as a suitable tester to identify useful lines in breeding programs. The testcrosses identified in this study can further be utilized in maize breeding programs as a source material for the development of aflatoxin-resistant PVA improved varieties, in addition to being recommended for release and registration after further evaluation across more locations and years.

There are many challenges in breeding maize for resistance to *A. flavus* infection and aflatoxin contamination. Increasing PVA in maize grain can be an approach for inclusion in integrated aflatoxin management programs composed of pre- and post-harvest practices, use of biocontrol agents, improved drying, optimal storage, and supplemented with appropriate policy and institutional actions to decrease contamination to the lowest possible. This study has shown that biofortified maize can significantly reduce aflatoxin contamination of maize products while providing a natural source of vitamin A supplement. Production and consumption of maize with reduced aflatoxin content and sufficient PVA will result in reduced diseases, increased income, and overall well-being of populations in affected regions.

## Data availability statement

The original contributions presented in the study are included in the article/**Supplementary Material**. Further inquiries can be directed to the corresponding authors.

## Author contributions

Conceptualization: AM and AO-B. Methodology: MM, A-RM, ID, AM, and AO-B. Supervision: AOA-M, BO, AM, and AO-B. Data analysis: MM, A-RM, and ID. Manuscript draft: MM, AM, and AO-B. Resources: MM, RB, AM, and AO-B. Manuscript review and editing: MM, AOA-M, A-RM, BO, WM, SM, ID, RB, AM, and AO-B. All authors contributed to the article and approved the submitted version
